# Epigenetic Regulation of Ferroptosis in the Liver

**DOI:** 10.34133/research.0323

**Published:** 2024-02-21

**Authors:** Scott J. Dixon

**Affiliations:** Department of Biology, Stanford University, Stanford, CA 94305, USA.

Ferroptosis is a non-apoptotic form of cell death defined by the iron-dependent accumulation of lipid peroxides [1]. Iron is an essential redox-active metal that can be found at the active site of several enzymes. Iron can also be found in a pool of loosely coordinated or “labile” iron within the cytosol [2,3]. Iron is thought to be necessary for ferroptosis by helping to catalyze the formation of reactive oxygen species (ROS), including lipid ROS, that promote the execution of ferroptosis [4]. Both iron-binding enzymes and the labile iron pool likely contribute to the execution of ferroptosis, depending on circumstances [5]. In mammals, complex homeostatic mechanisms ensure the appropriate amount of iron uptake necessary for survival while preventing the excess accumulation of iron that could be directly toxic or enhance ROS formation [6]; disruption of systematic iron homeostasis is therefore presumed to have potent effects on ferroptosis sensitivity throughout the body.

Our basic understanding of the ferroptosis biochemical mechanism has improved in leaps and bounds over the past decade. There has also been substantial progress in understanding how the aberrant activation of ferroptosis can contribute to or drive disease in many tissues of the body, including heart, kidney, and brain [7–9]. Advances in our understanding of how ferroptosis contributes to liver pathology has also been notable, but with many unanswered questions about iron and ferroptosis regulation in this tissue remaining [10]. A new study by Min and colleagues [11] integrates mechanism and disease, illustrating how the induction of ferroptosis in liver tissue in mice can be caused by disruption of the epigenetic regulator histone deacetylase 3 (Hdac3).

HDAC enzymes regulate gene expression [12]. Within the nucleus, HDACs catalyze the removal of acetyl groups from lysine residues found in histone proteins. This changes the accessibility of the associated DNA to the transcriptional machinery, altering mRNA expression. Hypoacetylation is generally associated with reduced mRNA expression. HDAC3 is one member of a large family of HDAC enzymes, which vary in intracellular localization and function. There is evidence that these enzymes are not entirely redundant but rather have specific functions in the cell and in whole organisms. Thus, for example, altered HDAC3 expression is specifically associated with pathological tissue damage to various organs including heart, kidney, liver, brain, pancreas, and lung [13]. In different contexts, HDAC3 appears to regulate disease-associated processes in unique ways. The advance presented in the current work is to show how HDAC3 disruption in the liver can result in pathology that is associated with altered iron homeostasis and the induction of ferroptosis.

The authors demonstrate that deletion of *Hdac3* specifically in hepatocytes results in widespread effects on iron homeostasis in many tissues of the body, including iron accumulation within hepatocytes themselves. Mechanistically, these changes are linked to reduced expression of *Hamp*, which encodes the iron-regulating hormone hepcidin. At the molecular level, loss of *Hdac3* expression causes a global increase in histone H3 lysine 9 (H3K9) acetylation, but interestingly not around the *Hamp* promoter itself. Rather, reduced activation of the Hippo pathway in liver cells is linked to lower *Hamp* expression. The Hippo pathway effector YAP can bind to the *Hamp* promoter, and in this research, lower promoter acetylation is associated with reduced *Hamp* expression. In any case, the liver-specific increase in iron levels in *Hdac3* mutants is sufficient to drive a ferroptosis phenotype that results in liver damage. Notably, treatment of *Hdac3* mutant animals with a specific inhibitor of ferroptosis, ferrostatin-1, or genetically reducing YAP expression, is sufficient to temper the tissue damage caused by *Hdac3* gene disruption.

These findings establish a complex relationship between liver-specific epigenetic regulation, systemic iron homeostasis, and tissue damage. While solving some mysteries, other new questions are raised by these results. For example, at the cellular level, what is the threshold for liver iron accumulation that leads to ferroptotic damage and cell death? The ability to systematically modulate iron levels in this tissue by manipulating Hdac3 and YAP pathway activity could provide a means to define where the threshold exists above and beyond which normal cells can no longer tolerate iron accumulation and ferroptosis susceptivity rises substantially. At the tissue level, it would be interesting to know whether a rise in iron levels alone is enough to predispose to ferroptosis, or whether other concomitant changes in gene expression occasioned by *Hdac3* disruption synergize with increased iron levels to modulate ferroptosis. For example, a detailed exploration of lipid metabolism in wild-type and *Hdac3*-mutant livers may reveal changes in the levels of specific polyunsaturated lipids [14] that, in combination with increased iron abundance, tend to predispose to ferroptosis. Alternatively, the expression of canonical anti-ferroptotic proteins such as Gpx4 or Fsp1 could be altered. More broadly, it may also be of interest to better understand whether modulation of YAP function could provide a general means to modulate ferroptosis. There is evidence that YAP signaling can promote ferroptosis in the cancer context, also in part by enhancing iron levels [15]. It may be that YAP signaling, activated downstream of *Hdac3* disruption or other stimuli, provides a general key for sensitizing cells to ferroptosis. Whether activating ferroptosis or enhancing sensitivity to this process is a desirable effect, as it may be in cancer, or one that is undesirable, as it certainly is in liver in response to injury, will very much depend on the context.
